# Isomaltulose-Based Stimulant Beverages Can Improve Postprandial Metabolic Responses Without Compromising Cognitive Benefits Associated with Caffeinated Energy Drinks

**DOI:** 10.3390/nu18071163

**Published:** 2026-04-06

**Authors:** Peter Michael Bloomfield, Nicholas Gant

**Affiliations:** 1 School of Exercise, Sport and Rehabilitation Sciences, University of Auckland, Auckland 1010, New Zealand; peter.bloomfield@auckland.ac.nz; 2 Centre for Brain Research, University of Auckland, Auckland 1010, New Zealand

**Keywords:** cognition, insulin, glucose, isomaltulose, sugar-sweetened beverages, energy drinks

## Abstract

**Purpose**: We hypothesised that cognition following consumption of an isomaltulose beverage would be comparable to that of an isoenergetic sucrose-based beverage, but the latter would attenuate post-ingestive metabolic responses. **Methods**: Thirty adults (15 males, 15 females) aged 21–44 years completed three experimental sessions, following at least 3 h fasting. Plasma insulin and glucose were measured in arterialised capillary blood 30 min after beverage consumption. Cognitive functions were assessed 45 min after beverage consumption using a computerised test battery; the primary cognitive performance outcome was a composite neurocognitive index score. Subjective symptoms were measured using questionnaires. Data are presented as the mean [95% confidence interval]. **Results**: Circulating glucose was greater after ingesting sucrose compared to isomaltulose and placebo beverages (sucrose: 7.3 [6.9, 7.7] mmol·L^−1^; isomaltulose: 6.3 [6.1, 6.6] mmol·L^−1^; and placebo: 5.3 [5.2, 5.4] mmol·L^−1^). Insulin rose to a greater degree with sucrose compared to isomaltulose (mean difference = 8.5 [2.4, 14.6] µU·mL^−1^, *p* = 0.005). Non-inferiority was shown between isomaltulose and sucrose for the composite neurocognitive index score (isomaltulose mean score = 0.931 [−2.3, 4.2]; sucrose mean score = 0.414 [−2.6, 3.5]). However, performance with the sucrose and placebo beverages was similar, limiting broader interpretation. The sensation of postprandial tiredness for isomaltulose was non-inferior to sucrose (isomaltulose mean score = −3.8 [−15.8, 8.2]; sucrose mean score = 0.1 [−10.9, 11.1]). **Conclusions**: A commercial stimulant beverage with isomaltulose as the energy substrate elicits substantial reductions in glycaemic and insulinaemic responses compared with an isoenergetic sucrose-based beverage, without compromising cognitive performance.

## 1. Introduction

Energy drinks are popular beverages for supporting cognitive function, typically containing caffeine and simple carbohydrates as the primary functional ingredients. However, the consumption of high-sugar beverages leads to rapid glycaemic and insulin responses, which are likely a contributing factor in the development of metabolic disease and the current obesity epidemic [1]. Although sucrose is the typical carbohydrate used in energy drink formulations, isomaltulose is a potentially favourable alternative. While sucrose and isomaltulose have equivalent energy densities, the latter is digested more slowly, provoking a lower glucose and insulin response [2,3,4]. Lower postprandial insulin and glycaemic responses are associated with improved insulin sensitivity, lowered risk of type 2 diabetes, and lowered risk of cardiovascular events [5]. Sustained delivery of energy via isomaltulose may help in preventing the “sugar crash” that can occur after consumption of a product high in sugar. Consequently, isomaltulose-based energy beverages could have a beneficial impact on the metabolic health of regular consumers, compared to typical sucrose-based beverages. Furthermore, isomaltulose may offer improved oral health outcomes due to its non-cariogenic property, whereby the glucose-fructose bond cannot be broken by most mouth bacteria [2]. This non-cariogenic property has a US Food and Drug Administration-approved health claim.

Caffeine and other stimulants do not play an important role in the provision of energy, but are the major functional ingredient in energy drinks, where acute ingestion improves components of cognition such as reaction time, information processing, alertness, and attention [6]. In order to be acceptable to regular users, an isomaltulose-based energy drink would have to provide similar cognitive benefits as a typical sucrose-based drink. Several studies have investigated the effects of isomaltulose on cognitive performance. Studies in adults show similar results between sucrose and isomaltulose on a range of cognitive measures, including executive function and working memory [7,8,9]. Two studies conducted in children aged 5–6 years suggested that some measures of cognition were improved following consumption of milk containing isomaltulose, compared to other sources of carbohydrate [10,11]. However, the participant characteristics in these studies are likely considerably different from those of regular energy drink consumers, so these findings must be interpreted with caution. Importantly, none of these studies have compared isomaltulose with sucrose in the presence of caffeine (i.e., a typical energy drink). We aimed to extend previous findings into a relevant real-world scenario using caffeinated energy drinks. Given the high global consumption of energy drinks and the current obesity epidemic, investigating potentially alternative ingredients for energy drinks that may promote improved metabolic responses is critical.

This study compared the effects of a sucrose-based caffeinated beverage with an isomaltulose-based caffeinated beverage that also contained a small amount of l-theanine. Specifically, we examined the effects of the drinks on cognition, plasma glucose, plasma insulin, mood, and impulsivity. We hypothesised that isomaltulose would result in a lower increase in blood glucose, compared to sucrose, and that this would be reflected by a lower insulin response. Furthermore, we hypothesised that cognitive performance and subjective mood after consuming an isomaltulose-l-theanine beverage would be non-inferior to the sucrose-based drink.

## 2. Materials and Methods

### 2.1. Experimental Design and Participant Characteristics

A pseudo-randomised, double-blind, placebo-controlled, crossover, non-inferiority trial design was used. Ethical approval was obtained from the University of Auckland Human Participants Ethics Committee (protocol number 018240, 21 December 2019), and the study aligned with the Declaration of Helsinki, except for prospective registration in a database. The study was retrospectively registered (ACTRN12626000213347 (https://anzctr.org.au/Trial/Registration/TrialReview.aspx?ACTRN=12626000213347, accessed on 19 February 2026)). Recruitment started on 4 April 2022 and ended on 3 October 2022.

Participants signed informed consent before taking part in the study. Thirty participants (15 males, 15 females) with a mean age of 25.4 years (range 21 to 44 years) and a mean body mass index of 24.2 kg·m^−2^ (range 19.8 to 38.5 kg·m^−2^) took part. Mean weekly caffeine intake was 1286 mg (standard deviation = 1261 mg, range 1 to 5364 mg), as estimated by a self-report questionnaire. Eligibility criteria required participants to be aged ≥18 years, willing to consume caffeine, and not be pregnant or breastfeeding.

**Figure 1:**
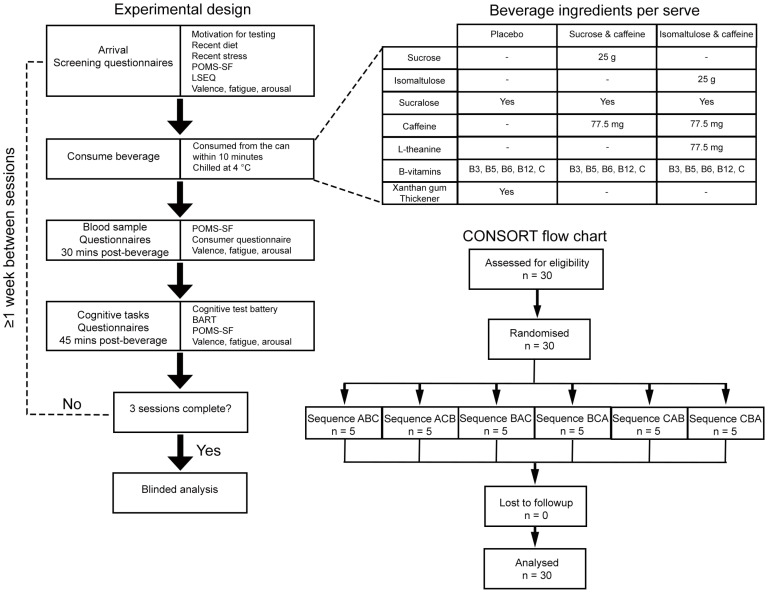
Flow chart displaying the experimental design, beverage ingredients, and CONSORT flow chart.

Three batches of drinks were used, each comprising placebo, isomaltulose, and sucrose drinks. The ingredients of the drinks are shown in Figure 1. The first ten participants received the placebo drink first (participants 1–10 formed the pilot study), participants 11–20 received the isomaltulose drink first, and participants 21–30 received the sucrose drink first. See Figure 1 for the CONSORT flow chart. For all three batches, the order of remaining drinks was randomised by an independent statistician using an online random number generator (https://www.sealedenvelope.com/simple-randomiser/v1/lists, accessed on 29 March 2022). Participants were randomised in blocks of 10 using a restricted permutation of treatment sequences, stratified by first-period treatment. Consequently, the design is fully counterbalanced over all possible sequences. In all cases, the participants were informed that all 3 drinks were fully randomised. The researcher conducting data collection and analysis was blind to the administration order of the two test solutions (sucrose and isomaltulose).

Participants completed three experimental sessions separated by approximately 7 days (Figure 1). Participants arrived at the laboratory after fasting for a minimum of 3 h and abstaining from caffeine on the day of testing. The final meal before fasting was uncontrolled and unrecorded by the researchers (consumed off-site). The fasting state was confirmed with a glucose test strip. Each drink was consumed within 10 min. All participants completed all sessions, and there were no adverse events. Participants received NZD 200 in supermarket vouchers as compensation for their time.

### 2.2. Measures

#### 2.2.1. Neurocognitive Tests

The test battery (Central Nervous Systems Vital Signs, CNS Vital Signs, LLC, Morrisville, NC, USA) was completed on a computer in an environmentally controlled laboratory 45 min after ingestion of the drink. This timing aligns with previous studies investigating caffeine [6] and isomaltulose [7] on cognition. All tests were administered by the same researcher and completed on the same computer (Lenovo V14-ADA laptop, 14″ HD, AMD 3020e, 8 GB RAM, 256 GB SSD, Lenovo, Beijing, China). Detailed instructions for each test were included in the battery, and the tests were self-paced. The following tests were included in the battery: verbal memory, visual memory, Stroop test, shifting attention task, symbol digit coding, continuous performance task, and four-part continuous performance task. The verbal memory test consisted of 15 words, presented for 1 s at 1 s intervals. Participants were required to memorise the words and then identify them when presented amongst 15 distractor words, immediately and approximately 25 min later, to assess immediate and delayed memory. The visual memory test was the same design but using geometric shapes instead of words. The Stroop test contained three parts. In the first part, participants responded as soon as a word appeared on the screen (red, blue, yellow, or green). In the second part, participants responded when the ink colour of the word matched the word. In the third part, participants responded when the ink colour and word did not match. The Stroop test assesses executive function and complex reaction time. The shifting attention task consisted of a single blue or red shape (circle or rectangle) at the top of the screen. Two other shapes would appear at the bottom left and right of the screen. Participants were given a command to match based on either shape or colour. This test assesses cognitive flexibility and executive function. The symbol digit coding consisted of a series of symbols that participants had to match to a list of numbers (e.g., 1 = # and 2 = *); this test assesses information processing and reaction time. The continuous performance task was a 5 min task where participants had to respond to the letter B appearing on the screen, but avoid responding when any other letter appeared. This test provides a measure of sustained attention, vigilance, and simple reaction time. The four-part continuous performance task examined sustained attention, reaction time, and working memory. Part 1 was a simple reaction time task, while part 2 was a choice reaction time task. Part 3 was a 1-back, where participants had to respond if two consecutive shapes matched. Part 4 was a 2-back, where participants responded if the shape matched the one presented before the previous shape (i.e., two shapes back). These seven tasks assessed 11 neurocognitive domains: composite memory, psychomotor speed, reaction time, complex attention, cognitive flexibility, processing speed, executive function, verbal memory, visual memory, working memory, sustained attention, simple attention, and motor speed. These domains were also summarised into a single neurocognitive index (NCI) score. For more information on how the domain scores are calculated in Central Nervous Systems Vital Signs, see https://www.cnsvs.com. There was no separate familiarisation session for the cognitive test battery, which we acknowledge as a limitation. However, the test battery included a practice version of each task that participants completed in each experimental session.

Impulsivity was measured using the BART, which is a computer-based task that rewards risky behaviour but penalises risk-taking beyond a certain point [12]. Participants used a mouse to inflate a virtual balloon for 30 consecutive rounds. Each successful pump earned 5 cents, but bursting the balloon lost any money for that round. Participants could opt to save the money in any given round (contributing to the grand total) and move on to the next round after one or more pumps. The number of possible pumps for each balloon was drawn from a normal distribution with a mean of 64, a standard deviation of 35, and a range of 1–128. Participants were told that the number of possible pumps was random for each balloon. The variables extracted from the BART were the number of adjusted pumps (the number of pumps from the balloons that were not burst only), the amount of money earned, and the number of balloons burst. Participants could earn a maximum of NZD 60 in any given trial. The final total was randomly selected from the three sessions and added to the participant’s compensation (i.e., total compensation possible was NZD 260).

#### 2.2.2. Mood Assessment

The Profile of Mood States—Short Form (POMS-SF) is a 30-word list describing feelings that may be experienced. Each word is rated from 0 to 4, for how the participant felt in that moment; 0 = not at all, 1 = a little, 2 = moderately, 3 = quite a bit, and 4 = extremely. Each word falls under one of five domains: depression, vigour–activity, tension–anxiety, confusion–bewilderment, and fatigue. An overall score for each domain was obtained by summing the scores for all words within each domain. Participants completed the POMS-SF on three occasions: upon arrival, immediately after the blood sample, and immediately after finishing the BART. Visual analogue scales (VASs) were used to record motivation for testing, self-assessed diet, self-assessed stress, affective valence, arousal, and fatigue. The first three questions were only asked on arrival to rule out confounding factors. Affective valence, arousal, and fatigue were measured upon arrival, immediately after the blood sample, and immediately after finishing the BART. A separate VAS was used to assess a set of consumer questions; these were only asked immediately after the blood sample.

#### 2.2.3. Blood Sampling

A 500 µL blood sample was taken via a finger prick 30 min after consumption of the drink. This timepoint is similar to that used previously and was selected on the basis of previous studies showing the peak plasma glucose concentration is approximately 30 min following sucrose or isomaltulose ingestion [7,9,13]. The hand was warmed in hot water for 5 min before the sample was taken. Samples were centrifuged at 3000 revolutions per minute for 10 min; the plasma was then pipetted into Eppendorf tubes and stored at −80 °C. Plasma insulin and glucose were measured using high-performance liquid chromatography.

#### 2.2.4. Triangle Test

A triangle test for sensory discrimination was used to determine if participants could identify differences between the sucrose and isomaltulose drinks. Participants were presented with three 30 mL samples, two of which were identical and one odd. Participants completed the test twice consecutively (once with 2 isomaltulose and 1 sucrose, and once with 2 sucrose and 1 isomaltulose) and rinsed the mouth with water between each sample. A total of 34 participants were recruited for the triangle test. Under the null hypothesis, the probability of correctly identifying the odd sample under chance alone was 1/3. Observed correct responses were compared against this probability using a binomial test, with a significance level of alpha ≤ 0.05.

### 2.3. Statistical Analysis

Based on a pilot study conducted in the first 10 participants, we found that the overall composite measure of cognition was improved by 8–10% by the sucrose and caffeinated beverage compared to the placebo (Cohen’s d effect size = 1.3). As caffeine is known to be particularly effective for improving attention [6], we used the simple attention data to calculate the required sample size. We recruited a total of 30 participants as a power calculation using the SampleSize4ClinicalTrials package in RStudio v2025.09.0+387 (R Core Team, 2019), with a margin of 4% and a mean difference of 0.2%, estimated that 29 participants would be required to demonstrate non-inferiority for simple attention (power = 0.8, alpha = 0.05).

Statistical analysis was conducted in Prism 9 (Graphpad Software, LLC, Boston, MA, USA). A linear mixed effects model with the general formula outcome ~ beverage + 1 | subject was used to determine if superiority was present in a particular situation (glucose and insulin data). Outcome was the outcome variable of interest, beverage was the factor for the drinks (3 levels: placebo, sucrose, and isomaltulose), and subject was the random effect of each participant. If a significant main interaction was detected, Tukey’s Honestly Significant Differences was used to make pairwise comparisons. To assess non-inferiority for the cognitive functions and subjective symptoms, we used 95% confidence intervals to compare the sucrose and isomaltulose drinks. For any given measure, if the lower confidence limit for the isomaltulose drink was above the lower confidence limit for the sucrose drink, non-inferiority was accepted. We opted for this approach as there was a lack of suitable margins against which to compare non-inferiority. This approach aimed to be more conservative than using a potentially inappropriate margin and to remove the risk of estimating margins based on previous studies. Unless otherwise stated, data are presented as mean (SD) or mean [95% confidence interval].

## 3. Results

### 3.1. Blood Glucose and Insulin

**Figure 2:**
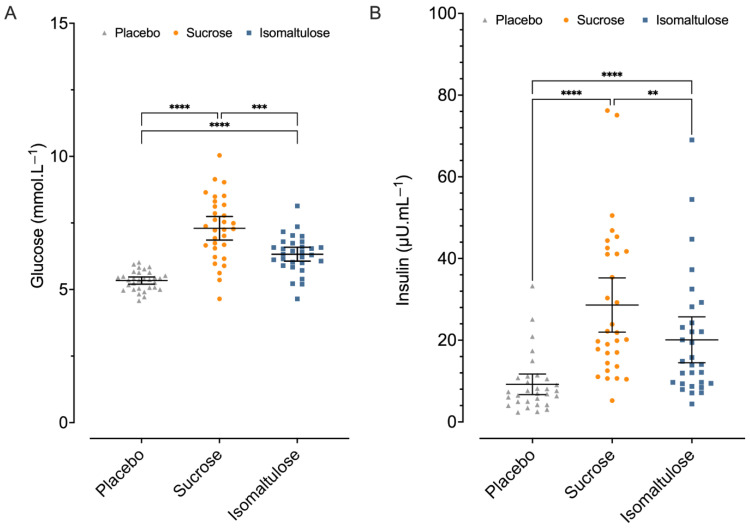
Glucose (**A**) and insulin (**B**) concentrations measured 30 min post-drink. Individual datapoints for *n* = 30 are presented with the mean and 95% confidence intervals. Triangles denote the placebo, circles denote sucrose, and squares denote isomaltulose. Pairwise comparisons are shown. ** denotes *p* ≤ 0.01, *** denotes *p* ≤ 0.001, **** denotes *p* < 0.0001. Data were analysed using a linear mixed effects model with Tukey’s HSD post hoc comparisons.

The mean time for collecting the blood sample for trials 1, 2, and 3 was 34 (3) min, 33 (3) min, and 34 (3) min post-drink. There were no significant differences between trial numbers (*p* = 0.26). Plasma glucose was significantly increased by both sucrose and isomaltulose (both *p* < 0.0001), compared to the placebo (Figure 2A). Furthermore, the increase in plasma glucose with sucrose was almost double that with isomaltulose (1.97 mmol·L^−1^ vs. 0.99 mmol·L^−1^, *p* < 0.0001). Compared to the placebo, sucrose elevated plasma insulin by 19.4 µU·mL^−1^ [13.0, 25.8 µU·mL^−1^] (*p* < 0.0001; Figure 2B). Isomaltulose increased plasma insulin by 10.9 µU·mL^−1^ [5.5, 16.3 µU·mL^−1^] (*p* < 0.0001). Insulin increased to a greater degree with sucrose compared to isomaltulose (mean difference = 8.5 µU·mL^−1^ [2.4, 14.6 µU·mL^−1^], *p* = 0.005; Figure 2B).

### 3.2. Cognitive Performance and Subjective Symptoms

**Figure 3:**
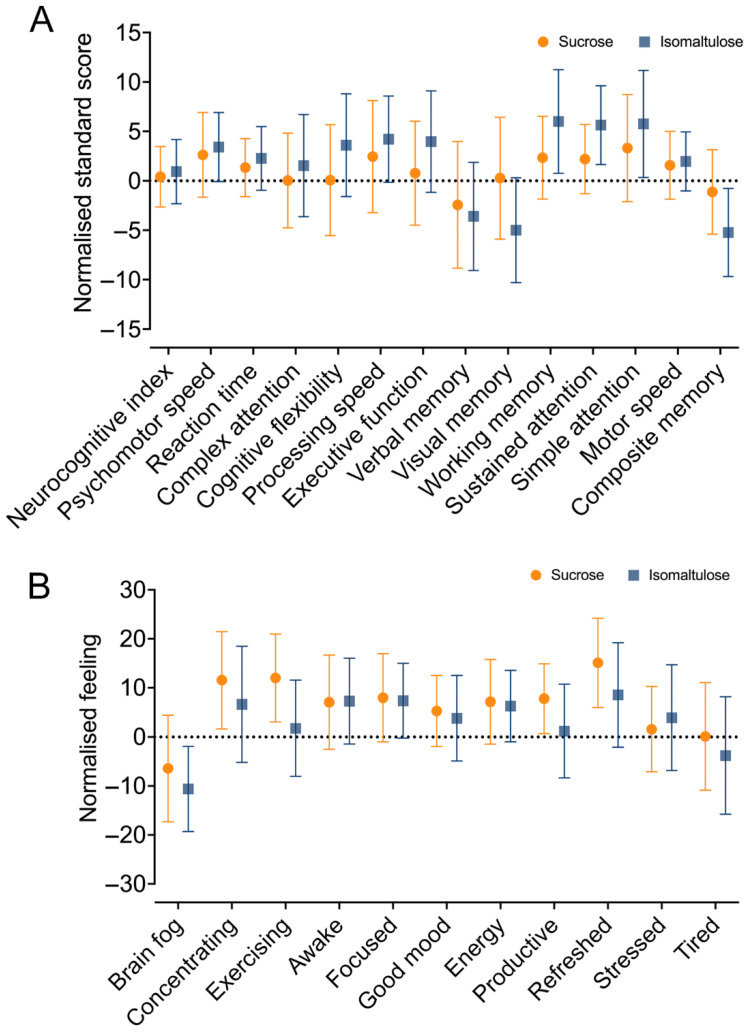
Normalised neurocognitive domain standard scores for each drink (**A**), and consumer questionnaire scores (**B**). Circles denote sucrose and squares denote isomaltulose. Data are presented as the mean with 95% confidence intervals for *n* = 30. For (**A**), domain scores for the isomaltulose and sucrose drinks were normalised to the placebo score. A score greater than zero indicates an improvement relative to the placebo, whilst a score lower than zero indicates worse performance relative to the placebo. For (**B**), a lower score is desirable for brain fog, stress, and tiredness; for the remaining feelings, a higher score is more desirable.

For each subject, the scores for the sucrose and isomaltulose drinks were normalised by subtracting the scores for the placebo drink; these scores are shown in Figure 3A. Non-inferiority was shown for the overall neurocognitive index score, psychomotor speed, reaction time, complex attention, cognitive flexibility, processing speed, executive function, working memory, sustained attention, simple attention, and motor speed (i.e., the lower confidence limit for the isomaltulose confidence interval was above the lower confidence limit for sucrose). The results for composite memory, verbal memory, and visual memory were inconclusive (i.e., the confidence interval for isomaltulose overlapped the lower limit for sucrose). When comparing across the three trials (trial 1, trial 2, and trial 3), a significant between-trial learning effect was present for all domains except composite memory, verbal memory, visual memory, simple attention, and motor speed. Importantly, comparison of the confidence intervals showed that the sucrose beverage did not significantly improve cognitive performance compared to the placebo beverage.

Results from the consumer questionnaire are shown in Figure 3B. A lower score is desirable for brain fog, stress, and tiredness; for the remaining feelings, a higher score is more desirable. Non-inferiority between sucrose and isomaltulose was shown for brain fog and tiredness. Equivalence between sucrose and isomaltulose was shown for awakeness, focus, and energy. Results for concentration, exercise, good mood, productivity, refreshness, and stress were inconclusive.

Affective arousal, fatigue, and valence were very similar between beverages. Arousal increased slightly over time with both the sucrose and isomaltulose beverages. Isomaltulose was non-inferior to sucrose at both the 30 min and post-test timepoints. Fatigue decreased slightly over time with both the sucrose and isomaltulose beverages; the non-inferiority results for both timepoints were inconclusive. Affective valence remained fairly consistent over time with both the sucrose and isomaltulose beverages; the non-inferiority results for both timepoints were inconclusive.

The POMS-SF scores were similar between sucrose and isomaltulose at all timepoints. Non-inferiority was shown for the confusion and tension domains at both the 30 min and post-test timepoints. Results for the depression, fatigue, and vigour domains were inconclusive.

### 3.3. Balloon Analogue Risk Task

**Figure 4:**
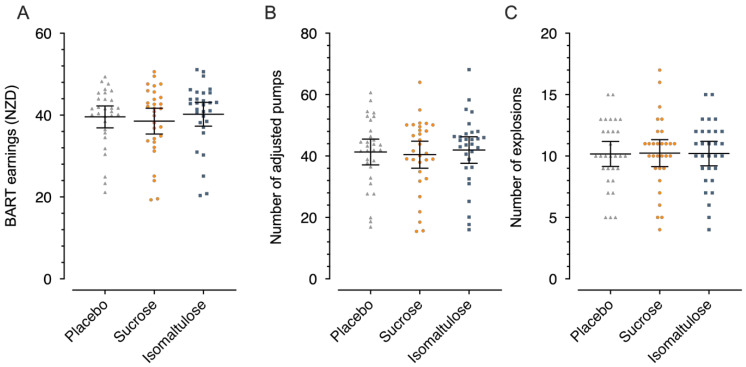
Task earnings (**A**), number of adjusted pumps (**B**), and number of explosions (**C**) during the balloon analogue risk task (BART). Triangles denote placebo, circles denote sucrose, and squares denote isomaltulose. Individual data are shown for *n* = 30 with a mean line and 95% confidence intervals.

Results for the BART were similar between beverages (Figure 4); however, the isomaltulose drink was non-inferior to the sucrose drink for all variables.

### 3.4. Triangle Test for Sensory Discrimination

Correct identification rates exceeded those expected by chance (1/3) in both trials (18/34, *p* = 0.014; 19/34, *p* = 0.006). These correct identification rates were significantly greater than those expected by chance, indicating a detectable sensory difference between the sucrose and isomaltulose beverages. However, participants did not have information about the ingredients of the beverages, and so the ability to detect any differences in the samples could not have affected performance on the cognitive tasks.

## 4. Discussion

We hypothesised that an isomaltulose-based energy drink would lower postprandial glucose and insulin levels and that cognitive performance assessed 45 min after ingestion would remain comparable to a sucrose-based drink. In support of this, our findings showed that isomaltulose produces a substantial reduction in plasma glucose and insulin appearance without negatively altering performance in several key neurocognitive domains and measures of subjective feeling. Non-inferiority between sucrose and isomaltulose was shown for all assessed cognitive domains except for composite memory, verbal memory, and visual memory.

Our cognitive performance findings are broadly consistent with those reported in previous studies [7,8,9], although the current study employed a non-inferiority design rather than traditional superiority testing. Across all domains demonstrating non-inferiority, effect sizes between sucrose and isomaltulose were small and consistently favoured the isomaltulose drink, highlighting that a lower glycaemic index does not impair cognitive performance. Importantly, the largest effect sizes (albeit still small) were for executive function, working memory, and attention; these domains may be the most desirable to target for energy drink consumers as they are involved in higher-level brain functions important for performance on many tasks (e.g., studying and driving). These results suggest that isomaltulose-based stimulant beverages offer a potentially suitable alternative to typical sucrose-based stimulant beverages.

There were comparable cognitive performance and mood states between energy drinks, supporting prior research that the rate of glucose appearance is not a significant factor in mediating mental performance [14]. However, this similarity across all three conditions (i.e., little effect compared to placebo) limits the extent to which effects can be attributed to carbohydrate type. In this context, the findings are more appropriately interpreted as indicating comparable cognitive performance across conditions, rather than a specific advantage of one carbohydrate source over another. Although the carbohydrate supplies the energy, caffeine is expected to be primarily responsible for changes in cognition. The lack of effect of stimulant beverages compared to placebo was unexpected, as this dose of caffeine is typically expected to improve measures of cognition. It is possible that the effect of trial order we observed may have masked the effect of caffeine.

Postprandial glucose and insulin levels were significantly lower after 30 min with isomaltulose than with sucrose. Although postprandial glucose and insulin levels were only assessed 30 min after ingestion, rather than using an area-under-curve analysis, these results are in line with previous research [9]. As glucose and insulin in arterialised capillary blood are typically higher than in venous blood, direct comparisons with studies utilising venous blood could be made with caution. The observed reduction in the glycaemic and insulinaemic responses is of a magnitude that may afford metabolic health effects for regularly consumed products [5]. Given the associations between glycaemic index, postprandial glucose levels, and markers of cardiovascular disease, as well as metabolic syndrome [15], isomaltulose-based beverages may provide a lower-risk option for frequent consumers of energy drinks, particularly those with pre-existing health conditions. However, as all our participants were healthy adults, further research in users with metabolic disorders or in different age groups should be conducted. The study was not powered to detect differences between sexes; however, the cognitive function scores were similar between males and females. Therefore, the results from this study are likely relevant to both, but larger studies would have to be conducted to confirm this.

There are several experimental considerations of this study that must be addressed. The pseudo-randomisation of the treatment order could potentially introduce period and recruitment order confounds. Consequently, it is not possible to fully separate the effect of treatment from period, recruitment order, or learning effects. However, since all participants were random volunteers with no knowledge of the drink ingredients, and the number of participants receiving each condition first was balanced across the entire sample (*n* = 10 per condition), period and recruitment order effects are still likely to be smaller than a learning effect due to practice over time. Including a separate familiarisation session, where participants completed the cognitive test battery at least once before data collection, may have reduced this learning effect.

There was a difference in l-theanine content between the sucrose and isomaltulose beverages; consequently, the effects of isomaltulose and l-theanine cannot be separated. Although l-theanine is thought to exert an antagonistic effect on caffeine and may impair cognition when taken alone [16], we saw no evidence for an effect on cognition or subjective symptoms. However, simultaneous ingestion of caffeine and l-theanine may improve some aspects of cognition, although the doses of caffeine and l-theanine used in the current study were considerably lower than those used previously [16]. The effects of l-theanine might be detectable if a larger dose of caffeine were used (i.e., regular consumers of multiple caffeinated beverages per day). Although we cannot rule out that the l-theanine could have offset potential negative effects of isomaltulose, we consider this unlikely, as there is no consistent evidence that isomaltulose would worsen cognition, compared to sucrose. It is important to note that, although the effects of caffeine on cognition are well established [6], the confidence intervals indicate that the sucrose-based beverage did not improve cognition compared to the placebo. It is possible that the learning effect we observed over the three trials contributed to the lack of effect. Moreover, as most participants were regular caffeine users, some may have been more desensitised to caffeine than others. Recruiting solely naïve users may have shown the expected difference between caffeine and placebo. However, naïve caffeine users are potentially less likely to consume energy drinks. We performed blood sampling at a single timepoint only, which is a less accurate reflection of the total insulin and glycaemic responses compared to multiple sampling and area-under-the-curve determination. The use of a single time point was chosen partly to reduce the effect of repeated blood sampling on subjects’ fatigue and mood responses, but also because a 30 min timepoint is appropriately supported by prior studies showing that peak glucose and insulin concentrations are attained approximately 30 min following consumption of both beverages [7,9]. However, it is possible that differences between beverages may be more apparent at later timepoints. Future studies incorporating multiple postprandial measurements would provide a more complete assessment of the temporal response. Participants also only fasted for 3 h, which could potentially influence baseline glucose and ensuing insulin response. However, due to the duration of the cognitive test battery, we considered it important that participants were not overly hungry when performing the cognitive tasks and were not fasted for longer than would be likely when consuming similar beverages.

## 5. Conclusions

This study demonstrates that a commercial stimulant beverage formulated using isomaltulose as the energy substrate elicits substantial reductions in glycaemic and insulinaemic responses compared with an isoenergetic sucrose-based beverage, without compromising mental performance factors. However, neither caffeinated beverage consistently improved cognition compared to a placebo, limiting broader generalisation. The amelioration of glycaemic responses of similar magnitude has been shown to afford metabolic health effects for regularly consumed foodstuffs. These findings hold relevance for the health of regular consumers of sugar-sweetened beverages and for commercial manufacturers.

## Data Availability

The original contributions presented in this study are included in the article/Supplementary Materials. Further inquiries can be directed to the corresponding author.

## Supplementary Materials

The following supporting information can be downloaded at: https://www.mdpi.com/article/10.3390/nu18071163/s1.

## Abbreviations

The following abbreviations are used in this manuscript:

BART	Balloon analogue risk task
NCI	Neurocognitive index score
POMS-SF	Profile of Mood States—Short Form
VASs	Visual analogue scales
